# Barriers and facilitators to optimal sepsis care – a systematized review of healthcare professionals’ perspectives

**DOI:** 10.1186/s12913-025-12777-8

**Published:** 2025-04-24

**Authors:** Lea Draeger, Carolin Fleischmann-Struzek, Sabine Gehrke-Beck, Christoph Heintze, Daniel O. Thomas-Rueddel, Konrad Schmidt

**Affiliations:** 1 Jena University Hospital, Institute of General Practice and Family Medicine, Friedrich Schiller University Jena, Jena, Germany; 2 Jena University Hospital, Institute of Infectious Diseases and Infection Control, Friedrich Schiller University Jena, Jena, Germany; 3 Jena University Hospital, Center for Sepsis Control and Care, Friedrich Schiller University Jena, Jena, Germany; 4 Institute of General Practice and Family Medicine, Charité University Medicine, Berlin, Germany; 5 Jena University Hospital, Department of Anesthesiology and Intensive Care, Friedrich Schiller University Jena, Jena, Germany; 6 Department of Anesthesiology, Montefiore Medical Center, Albert Einstein College of Medicine, New York, NY USA; 7 Institute of General Practice, Faculty of Health Sciences Brandenburg, Brandenburg Medical School Theodor Fontane, Brandenburg, Germany

**Keywords:** Sepsis, Septic shock, Healthcare providers’ perspective, Care processes, Barriers to optimal care

## Abstract

**Background:**

Despite therapeutic advances, sepsis remains a global burden. Shortcomings within the healthcare system that inflate morbidity and mortality rates are instructive in this regard. This review aims to provide a qualitative synthesis of literature related to healthcare providers’ perspectives on sepsis care, emphasizing perceived factors that impact the adequate care of septic patients and sepsis survivors.

**Methods:**

In February 2023, we conducted a systematized search approach using the PubMed database.

**Results:**

Of 114 articles found in the PubMed database, 37 were included. A further 13 articles were identified by manual search. Healthcare providers highlighted a variety of dysfunctional and functional processes with an impact on sepsis care. Six domains were identified, related to the underlying disease, the patient, the provider, the guidelines, the healthcare system, and the collaboration among providers. Of note, providers’ level of knowledge and a lack of communication between disciplines and/or sectors were reported as shortcomings in each phase of the care pathway (prevention, recognition, treatment, transitions of care, and aftercare).

**Conclusions:**

This review suggests that, without limitation, interventions that provide continuous provider education as well as standard communication channels between interdisciplinary and intersectoral providers have great potential to improve structural deficiencies in sepsis care.

**Graphical Abstract:**

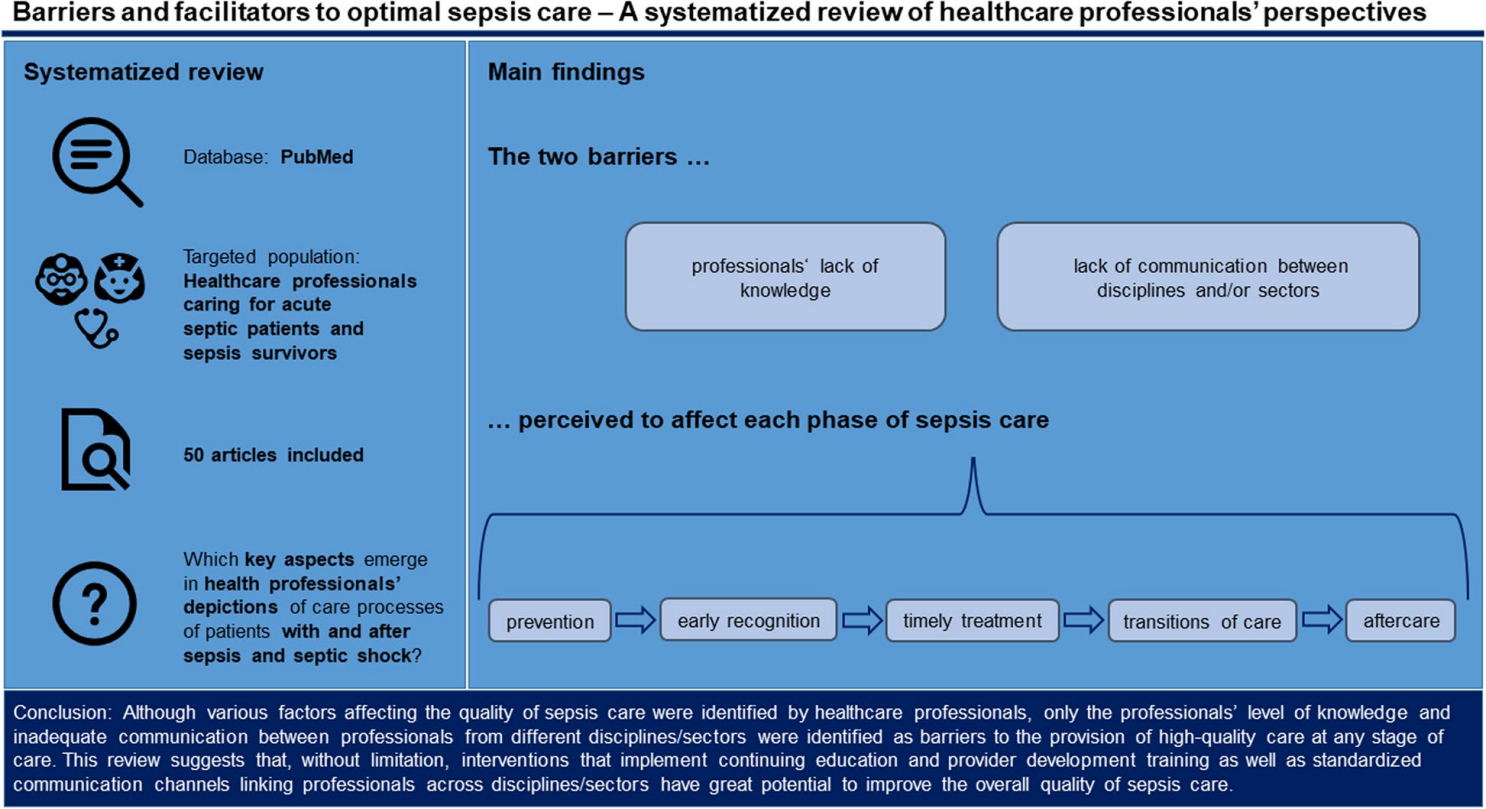

**Supplementary Information:**

The online version contains supplementary material available at 10.1186/s12913-025-12777-8.

## Introduction

Sepsis is defined as a life-threatening syndrome characterized by acute organ dysfunction due to an infection. 20% of all deaths globally are estimated to be associated with sepsis [1]. The condition poses a substantial challenge to healthcare professionals of all disciplines. Beyond high acute mortality [2, 3], sepsis is associated with an increased likelihood of 30-day rehospitalization [4, 5] and decreased chances of independent living 6 months after diagnosis [6]. As a time-sensitive disease, early recognition and rapid escalation of therapy are of paramount importance for survival and the clinical course [7]. However, adherence to evidence-based care bundles and guidelines remains incomplete, particularly outside the intensive care unit (ICU) [8, 9]. Further clarification is needed to understand the barriers to translating evidence into clinical practice. In addition, sepsis can lead to the so-called post-sepsis syndrome. This affects up to three out of four sepsis survivors and manifests with new or worsening physical, psychological, and/or cognitive symptoms [10] that often persist for months or even years after the index illness [11, 12]. Thus, in the long-term, sepsis is linked to a reduced health-related quality of life [13], increased healthcare utilization, and consequently, to an immense increase in healthcare expenditures [14]. Despite the magnitude of long-term sequelae, specific aftercare programs are still lacking [15]. Evidence of existing post-ICU services’ positive impact on survivors’ health is limited [16]. Therefore, the aim of this systematized review [17] is to synthesize relevant literature investigating healthcare providers’ perspectives on sepsis management in their everyday work and to elaborate barriers as well as facilitators to adequate care.

## Methods

A systematized literature review was conducted in PubMed in February 2023. A systematized review complies with many, but not all criteria of a systematic review process, therefore stopping short of a systematic review. The literature synthesis is performed narratively with a tabular supplement [17]. The search strategy was developed iteratively by an interdisciplinary team consisting of a psychologist and a physician, supported by a specialist from the Thuringian University and State Library. Relevant search terms were identified (1) deductively based on the study objective and (2) inductively based on topic-related terms (determined by prior literature screenings) that appeared to be common in the existing literature on sepsis. The final search strategy is listed in the supplementary material (see Additional file 1 for the complete search string).

Eligibility was assessed based on the following key inclusion criteria:

- assessment of health professionals’ perspectives on the management of sepsis or septic shock in adults with no limitation to discipline;
- application of quantitative, qualitative as well as mixed-method approaches;
- conduct in high-resource settings. As part of the AVENIR project, this restriction was made because this review services to inform qualitative interview studies about barriers and facilitators to optimal sepsis care among healthcare providers from the high-resource setting Germany.

We excluded studies that (a) described highly specific (intensive care) treatment practices or laboratory research, (b) were not written in English or German, (c) had no full text available, and (d) had been published prior to the year 2000 for reasons of contemporary relevance.

**Fig. 1:**
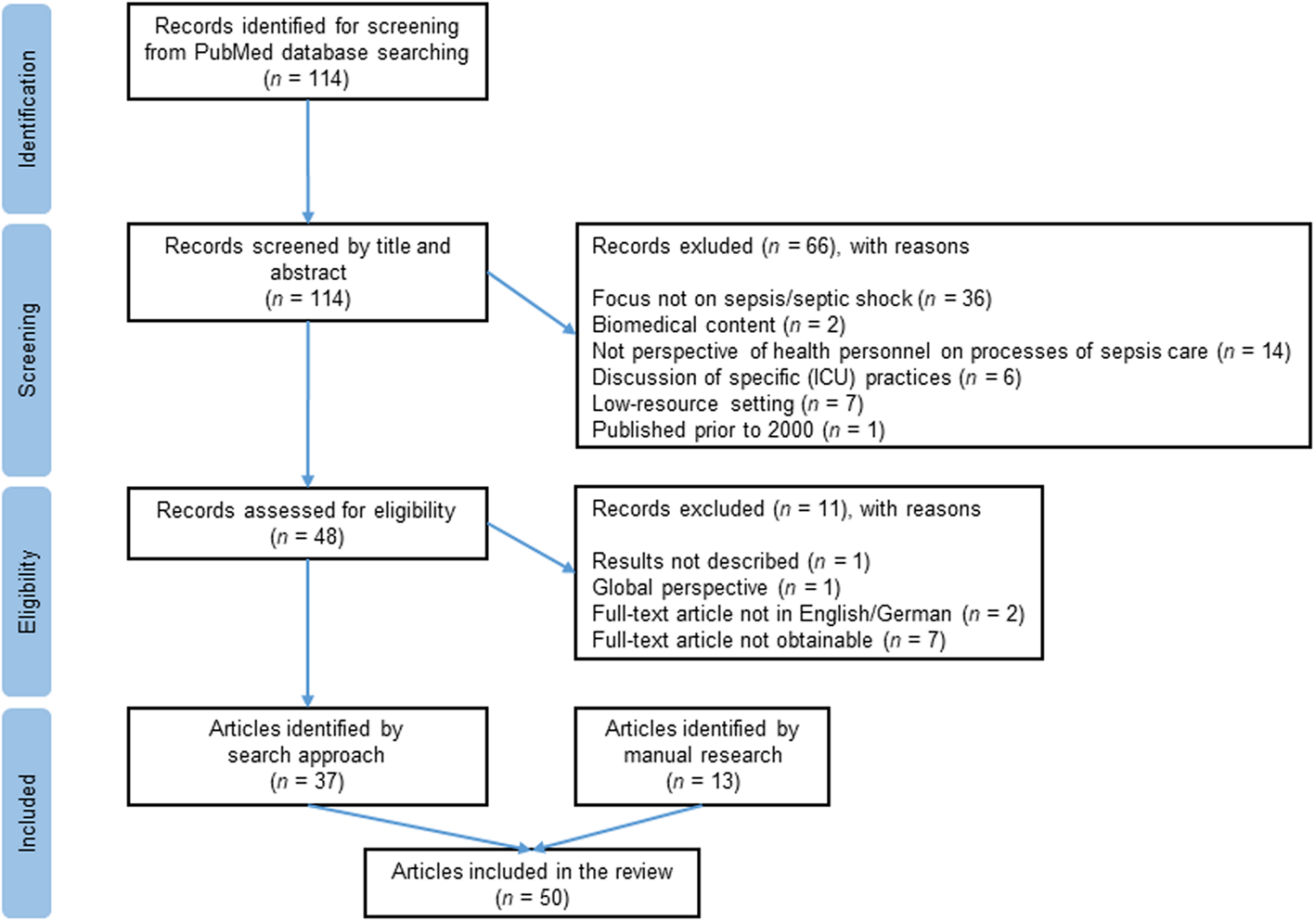
PRISMA flow diagram of included studies

This review applied a qualitative systematized data review approach by using a comprehensive systematic search strategy [17, 18]. The selection process at the title and abstract level resulted in 114 articles, of which 37 were eligible for inclusion (Fig. 1). Additionally, the bibliographies of the retrieved articles were scanned for further studies relevant to the review, and a manual search was performed. A total of 13 articles found through the manual search were also identified as eligible for inclusion, and 50 articles were included in this review in sum. The screening and selection of the literature were carried out by one researcher with consultative support.

One reviewer collected data from each article. Relevant outcomes were self-reported knowledge, practices, attitudes, perspectives, and opinions. Then, studies were grouped thematically according to the following overarching categories: (1) Prevention, (2) Early recognition, (3) Timely treatment, (4) Transitions of care, and (5) Aftercare. Study summaries were re-read and analyzed to inductively identify related information and common categories and codes that could be merged and narrowed down, while focusing on barriers and facilitators to optimal care. Using this non-linear approach in the style of meta-aggregation [19], information was interwoven to create a cohesive thread. Descriptive information of the included full-text articles is summarized in the Additional file 2.

### Risk of bias

As a structured critical appraisal of the retrieved articles, three checklists were used to assess study quality: the JBI Checklist for Analytical Cross-Sectional Studies [20] for quantitative studies, the CASP Checklist [21] for qualitative studies, and the MMAT [22] for mixed-methods studies. For the CASP Checklist, only the first 9 of the 10 questions were used. Since the 10th question is phrased openly and not in a yes/no format, it was not included in the appraisal. The individual rating of each item is presented rather than an aggregated score, which does not allow inferences to be drawn about each item (see Additional files 3, 4, and 5).

## Results

The 50 studies of this systematized review included participants from four continents across fourteen countries, being Australia (*n* = 2), Canada (*n* = 3), China (*n* = 1), Denmark (*n* = 1), England (*n* = 6), Germany (*n* = 7), Ireland (*n* = 1), Japan (*n* = 1), the Netherlands (*n* = 2), Poland (*n* = 1), Scotland (*n* = 3), South Korea (*n* = 1), Sweden (*n* = 1), and the United States of America (USA) (*n* = 20). Of these, 20 were quantitative, 18 were qualitative, and 12 used a mixed-methods approach. The quantitative studies mainly gathered information using questionnaires. Sample size varied between *n* = 14 and *n* = 1240. The qualitative studies mostly applied semi-structured interviews and focus groups. Sample size varied between *n* = 8 and *n* = 43. Studies using mixed-methods approaches predominantly combined questionnaires and interviews with sample sizes ranging from *n* = 11 to *n* = 706 participants.

### Prevention

Only one of the included studies examined the healthcare providers’ experiences with the prevention of sepsis. Primary care providers (PCPs) identified physician-, healthcare system-, patient-, and context-related barriers to the implementation of post-splenectomy sepsis prevention measures focusing on vaccination [23]. Concerning the barriers relating to PCPs themselves, knowledge gaps, as well as uncertainties about risks specific to asplenia, concurrent treatments that are contraindicated, and the post-discharge vaccination sequence were reported. Further, PCPs described updated vaccination recommendations and the scarcity of routine care for asplenic patients as impeding proper prevention. Apart from this, misleading information in the hospital’s discharge letter and the hospital’s failure to call patients’ attention to subsequent primary outpatient care were perceived as shortcomings at the interface between hospital and outpatient care. Perceived patient-attributed barriers were lack of awareness, comorbidities, and concerns about the late effects of vaccination. On the contextual side, PCPs indicated that delivery shortages of vaccines and demanding documentation requirements hindered implementation [23].

### Early recognition

Nineteen articles described healthcare providers’ insights into processes of early recognition of sepsis.

#### Knowledge

Emergency medical service (EMS) providers attributed great importance to sepsis care [24] but indicated gaps in their knowledge of sepsis criteria and relevant clinical signs [24–26]. Only a minority of them asserted confidence in recognizing a septic patient during the transport to the hospital [26]. Paramedics were the most aware and knowledgeable regarding diagnostic criteria among EMS providers [27]. Similarly, a majority of emergency department nurses and resident physicians possessed narrow sepsis knowledge [28–30]. Emergency nurses stated both the need and the desire to learn more [29]. Also on medical and surgical wards, the variability of knowledge regarding diagnostic criteria among registered nurses and residents has been described as a safety issue [31].

#### Barriers to timely diagnosis

Besides a lack of crucial knowledge and training [31, 32], further barriers hindering early sepsis detection described by providers of various disciplines included the availability of physicians on the general ward, time-limited staff-patient contact, the complexity of the disease [32], and a lack of consistency in detecting non-specific warning signs [32, 33]. Additionally, the lack of necessary equipment to evaluate for infection, such as thermometers on EMS units [33], and high patient acuity and volume were perceived to hamper recognition as they diverted resources away from the detection process [28]. Besides, lack of insight into patients’ baseline status, especially in non-vocal patients, delay in recognition by less qualified junior physicians due to lack of supervision by experienced physicians, and task-oriented rather than coaching-oriented actions by supportive nurses (so-called relief nurses) have also been reported to limit early recognition of sepsis. The coaching role of support nurses aims to assist other nurses in the development of skills to reflect critically on patients’ condition changes and to take action themselves, whereas purely task-oriented actions provide less teaching value [31]. In terms of further aids of recognition, professionals of various training levels and specialties recognized the importance of blood culture sampling as a diagnostic tool but did not consistently adhere to practice guideline recommendations – both in terms of sampling frequency and sampling performance [34]. Healthcare professionals rated the unawareness of the guidelines’ content and time constraints during everyday work as the main reasons for poor application. Regular training as well as more time in their work for continuing medical education were considered to be the most significant for improvement [34]. Generally, the ability to seek advice and intercollegial supervision and reflection were perceived as empowering and conducive to patient safety [28, 35].

#### Electronic early warning systems

Hospital leaders appealed for the establishment of clinical decision support systems for the early detection of sepsis. Disagreement prevailed as to whether clinical decision support has the predictive potential to provide clinically relevant specificity [36]. Frequent false-positive alerts [37] and consequential alert fatigue [38], perceived low precision [39], lack of perceived efficacy in detecting critically ill patients, lack of patient-centeredness and algorithms’ transparency [40] as well as high complexity [36, 38, 40] were voiced by some professionals as reasons for poor acceptance and trust. Nurses and acute care physicians (ACPs) viewed the machine learning-based systems as playing a more supportive and partnering role in diagnosis [39, 40]. The alerts were seen as complementary to human work, with the ultimate competence and responsibility for clinical decisions ascribed to the provider [39, 40]. Clinical expertise, intuition, and the visible cues of direct bedside interaction with the patient were considered nurse- and physician-specific advantages over support systems [39]. Predictive information was valued, for instance, when the data at hand were diffuse and the course of action was difficult to predict as well as when specific interventions were attached to the alert [37]. Understanding how the system works, the direct experience with the system, external studies validating its effectiveness, recommendations from colleagues and experts, and the integration of own recommendations into the tools’ operation were mentioned as helpful in building trust [39]. Further suggestions included minimizing the alert frequency by optimizing the threshold, providing explanations of the alert content, integrating the alert into the workflow by avoiding hard stops, and enhancing buy-in by providing direct feedback [36]. Nurses favored alert interventions that were predicated on an established treatment protocol (e.g., blood sampling) and focused on the patient’s clinical condition as a whole rather than on predefined thresholds of regulatory guidelines [41]. The utility of prediction algorithms offers an indication to call the medical emergency team [40] and has been addressed to be higher for junior and less experienced staff [37, 40]. Evidence indicated that the evaluation of the alert benefits and the systems’ impact on clinical care processes were partly dependent on the occupational group making use of it [38].

#### Referral to hospital

Both PCPs and emergency nurses pointed out that the decision-making process of referring suspected septic patients from primary care to the hospital and that sepsis recognition in clinical practice did not solely rely on vital parameters [28, 42]. In particular, general appearance, patient history, physical examination [42] as well as gut feeling/instinct [28, 42] were exemplified to guide diagnoses. A significant proportion of PCPs voiced feeling uncertain in their referral decisions [42]. Notably, ambulance service clinicians’ (registered nurses and emergency medical technicians) assessment of patients with suspected sepsis was largely driven by previous experience with similar patient cases. Moreover, they doubted the general usefulness of guidelines, which were deemed narrow when assessing complex and ambiguous clinical presentations [35].

### Timely treatment

Twenty-eight articles depicted healthcare providers’ insights into acute treatment of sepsis.

#### Prioritization of sepsis

EMS directors revealed that sepsis was attributed the lowest priority on a list of seven medical initiatives (including, but not limited to, cardiac arrest, ST-elevation myocardial infarction, and stroke) [33]. This low prioritization was also partially reflected in ACPs’ communication of sepsis-related health risks to patients. According to ACPs, minimizing the communicated risks of neutropenic sepsis served to avoid distressing patients and to increase adherence to important anti-cancer treatments which could cause this form of sepsis. Conversely, downplaying the disease’s seriousness may delay patient reporting of symptoms and subsequently delay management [43].

#### Barriers to timely treatment

The vast majority of prehospital care providers affirmed feeling confident and possessing the required skills to administer fluid therapy to patients with sepsis and septic shock. Especially clinical intuition strongly influenced therapy decisions. Uncertainties about whether the patient needs fluid and about the appropriate fluid volume were voiced. Many prehospital care providers criticized the lack of research and evidence on prehospital fluid therapy. Professionals expressed an interest in more education [44] and viewed sepsis protocols as the most purposeful resource to improve patient outcomes [45, 46]. Yet, in particular specialties outside of anesthesia, such as general surgery and general medicine, voiced to poorly adhere to protocols of time-sensitive therapy [47]. Professionals disclosed a fair level of guideline compliance in the ICU and a poor level of guideline compliance outside the ICU, for which varying levels of knowledge were discussed as underlying [48]. Low adherence might be due to the complexity of existing protocols [49, 50], which require sequential and interdependent steps of coordination, collaboration, and communication among multidisciplinary staff [49]. Protocols were also judged to be non-intuitive, resource-intensive [50], overloaded with information, and not provider-friendly [51]. Protocol-initiated care was often perceived as not meeting the individual needs of patients [45]. Healthcare providers criticized a strong focus on clinical documentation, which was perceived to not necessarily benefit the patient [50].

In general, delays in sepsis treatment were reported to be caused by the complexity and vagueness of how sepsis manifests itself [33, 49, 51, 52] as well as workplace-related factors given the high resource demands of sepsis care [29, 52, 53]: Explicitly listed were, for example, immense time pressure [49, 51], busy/heavy workload, high patient acuity [28, 49, 51, 54], competing demands requiring attention [49, 51], interruptions in the workflow [49], staffing shortages of nurses as well as physicians [28, 46, 52–55], delays in interventions by nurses (unclear whether due to, e.g., time demands imposed on nurses or a lack of practical skills) [55], equipment/treatment unavailability [28, 32, 54, 55], and time-consuming transfers of patients (to other wards) [32, 49]. Provider-related obstacles, such as knowledge gaps [24, 29, 30, 47, 48, 53, 55], the level of individual experience and assertiveness [28, 31, 32, 51], training [33, 54], the range of clinical skills [47, 49, 51, 52, 54], provider engagement and their negative attitudes towards protocol use [33, 45], fear of harming the patient in the absence of an established diagnosis [46], fear of the septic patient in general [53], and insufficient prioritization of treatment of severe sepsis and septic shock [28, 32] were also perceived to impede prompt escalation of care. Further, the operational hurdle of nurses’ lack of authorization, as manifested by their dependence on physician prescriptions and orders [32, 49, 51], uncertainty about the chain of command [31], physician unavailability [53, 54], and delays in prescriptions [54, 55] and laboratory results [32, 53, 55] were stated to slow down the escalation of therapy too. Professionals associated these time losses with a more task-oriented and less holistic assessment, which was described as reducing the degree of critical thinking and clinical reasoning [28].

#### Improvement strategies

To enhance prompt and uninterrupted escalation of care and minimize coordination and task-switching, the establishment of a schooled response team that retains full responsibility for therapy initiation and completion has been suggested [49]. This is assumed to reduce distractions, practice interruptions, and the need to unconsciously switch from one task to another. Therefore, error rates can be minimized [56, 57]. Granting nurse-initiated procedures without physician approval and prescription [32, 51, 54], holding the knowledge of sepsis triggers, being familiar with the respective bundles [46], and education and training on how to handle non-standard situations [46, 54] were also cited to improve timely management. Sepsis screening tools [54], perceived effectiveness of the respective protocols, and the perception that benefits of care escalation outweigh potential risks [46] as well as feedback, support, and advice from experienced colleagues and peers were also perceived to facilitate prompt care escalation [28, 46, 54].

#### Antibiotic stewardship

Although ACPs (critical care and infectious disease physicians) disagreed on which medical specialty should be responsible for antimicrobial stewardship, they perceived transdisciplinary collaboration in the ICU as highly desirable [58]. Interestingly, junior physicians perceived an imbalance in their responsibility to start, but not to review or stop infection management. They indicated that feedback from senior physicians regarding their prescribing decisions was often lacking, provoking frustration, and undermining learning gains in infection management [59].

Clinicians’ decisions on antibiotic treatment initiation in patients with suspected sepsis were influenced by both infection-related and provider-related factors [60, 61]. Clinical decision-making behaviors were studied in a standard model-based approach that estimated treatment thresholds by a sample of clinicians from three institutions for eight clinical vignettes. The decision to initiate antibiotic administration varied by the probability of infection and illness severity as well as by provider specialty and clinical experience [61]. More precisely, high severity of sepsis predicted low thresholds for the initiation of treatment, as withholding treatment would result in more serious health consequences. When the severity of sepsis was less apparent, however, ACPs depended on a high probability of infection for the administration of antibiotics. The specialization in infectious diseases predicted high treatment thresholds, whereas specialization in emergency medicine predicted low treatment thresholds. Less practical work experience seemed to be associated with low thresholds, and more practical work experience with high thresholds. While low thresholds may minimize mortality and other harmful consequences for health, they have also been discussed as increasing harms of treatment, such as bacterial resistance [61]. Prescribers reported that their decisions of prescribing were based on clinical risk assessment, considering both the potential consequences of inappropriate prescribing and inappropriate withholding of antibiotics. Sepsis was considered to be the most harmful consequence arising due to withholding [62].

From the perspective of nurses, a very high patient workload [63], lack of/inadequate availability of antibiotics on the unit [64], lack of awareness that intravenous antibiotics were available on / ordered to the unit [63, 64], and intravenous line access issues [63, 64] functioned as important barriers to timely antibiotic therapy. ICU physicians disclosed that their knowledge of antibiotic pharmacokinetics and pharmacodynamics was deficient. Research has shown that the level of knowledge may depend on hospital type and work experience [65].

### Transitions of care

Nine articles addressed providers’ descriptions of teamwork and patient transitions within and across sectors, which affect both early recognition and timely treatment.

#### Lack of communication

Interprofessional barriers such as failures in communication and collaboration, particularly between ACPs and nurses [31, 49, 53], hostile/intimidating work environments characterized by little respect [28, 53], interdisciplinary conflicts [51], and poorly coordinated patient handovers to another unit or sector [32, 55] were specified. In this context, interdisciplinary and interprofessional providers identified handover difficulties between the emergency department, the general ward, and the intermediate care unit/ICU. Medical histories and anamneses with incorrect content were named as prehospital sources of error, as was the absence of the responsible physician of the emergency department within patient handovers from the ambulance service to the emergency department. As to transitions from the emergency department to the general ward, healthcare professionals indicated that the low capacity of available hospital beds frequently delayed handovers. For handovers between the general ward and the ICU, the lack of staff for transportation, the absence of physicians in general, and long latencies of laboratory results as well as other diagnostics were identified as further causes of delay. The lack of communication and therefore information about the urgency with which to treat septic patients as well as different heterogeneous documentation systems characterized all transitions [32].

#### Improvement strategies

Healthcare professionals have suggested several strategies to overcome some of the aforementioned barriers: mandatory training sessions, guiding checklists and posters, reviews of septic patient cases who received substandard care, and the establishment of standard operating procedures have been proposed [32]. An experienced and knowledgeable physician should undertake emergency department triage. In addition, a physician as a central point of contact for all wards has been discussed as useful for timely response to emergencies [32]. To minimize information loss, the presence of a physician at the point of transition to another ward, completion of a sepsis checklist for all newly admitted patients, and personal verbal instructions were suggested [32]. Similarly, healthcare providers discussed that the interface between primary care and hospital, in particular, could be improved through feedback to facilitate reflective learning, effective communication pathways, early warning scores, revised electronic templates for recording physiologic parameters, education about sepsis as well as the completion of an electronic summary of patients’ clinical and social information compiled by the patient’s PCP and made available to other medical parties [66]. In addition, the implementation of an in-hospital quality improvement (QI) team was explored as a way to improve performance. QI team leaders listed five areas of supportive conditions for QI-related change, being (1) the availability of external support, such as from change counselors, (2) the interdisciplinary composition of the team, (3) positive staff characteristics, such as commitment and awareness/knowledge, (4) generally supportive structural conditions, such as adequate time and personnel resources, and (5) information about process changes circulating among staff, for example, through staff rotation [67].

### Aftercare of sepsis survivors

Seven articles provided healthcare providers’ insights into related processes of sepsis aftercare.

#### Information flow

In the field of critical care, patients’ complex long-term sequelae have been well known. Disability and weakness, psychiatric pathologies, and cognitive dysfunction were cited as the most frequently occurring issues [68]. Nevertheless, the potential challenges and changes in patients’ lives associated with these sequelae were not consistently communicated to sepsis survivors, their families, and their PCPs [68–70]. While mutual and effective information sharing between care providers was seen as beneficial to patient care by ACPs and PCPs, ACPs tended to initiate contact with PCPs to obtain information (e.g., patient’s background, medical history, regular medications and/or allergies, details leading to the current illness) rather than to share information (e.g., specific diagnosis of admission, length of stay, the severity of illness, patient death). ACPs mentioned structural barriers, such as time constraints, perception of low priority, and difficulties in establishing contact with the PCP, as contributing to the low information sharing about an ICU admission [69]. Consistent with the ACPs’ descriptions, PCPs expressed communication breakdowns on the hospital side that left them uninformed about their patients’ acute septic phase and uninvolved in treatment decisions made within the hospital [70]. They reported receiving information about the ICU stay mainly from patients and their relatives, who they did not always consider as reliable messengers [69]. They also criticized the incompleteness of the information presented in the general discharge summary [69, 71], as technical information typically outweighed clinical information [69]. As a result, they often felt poorly prepared for patient visits where hospital information was appealed and often followed unspecific interventions of aftercare [69]. Both ACPs and PCPs agreed that an unplanned admission to or a discharge from the ICU and a patient death in the ICU should initiate a two-way information flow. Information sharing at ICU admission should be brief and to the point, whereas reporting at discharge should be more explicit, providing information about why the admission occurred, what the consequences were, and what to target in future treatment. Contact at admission should not only serve to inform the PCP about the critical illness but also to provide ACPs with patient data relevant to early patient management [69].

#### Inpatient-to-outpatient continuity

Absence of care immediately after discharge was described by various providers. Care coordination between inpatient to outpatient services that offered diagnostics, counseling, psychological aftercare, targeted referral to specialists, and relatives’ involvement in the aftercare process was cited as a potential solution. The most frequently described challenge was the direct collaboration of different disciplines in the fragmented pathways of care [71]. While the implementation of best practices for sepsis recovery varied greatly, social support and medication management were most frequently adopted. Physical recovery and adaption as well as emotional support for sepsis survivors and their families were inconsistently offered [68, 72].

#### Primary care

Providers across all sectors assigned a key role to PCPs in both the aftercare process of critically ill patients and in care coordination [69, 71]. When asked about their experiences in caring for post-sepsis patients, PCPs emphasized the continuity of care and their good relationship with patients, characterized by detailed knowledge of the patient’s medical history, social background, personality traits, and illness coping mechanisms. PCPs acknowledged the impacts of sepsis sequelae, such as general weakness, low functioning, and overall diminished quality of life leading to stress for both the affected individuals and their families [70]. Some PCPs reported to have limited time, knowledge, experience, and skills to adequately support patients who underwent a prolonged ICU stay [69, 71].

External factors, such as limited access to further supportive care services (e.g., physiotherapy and psychotherapy) [69, 73], and patient-related factors, such as limited cost coverage [73, 74], were also perceived to impede the management of post-ICU impairments. Many PCPs felt that filling patient education gaps would facilitate the aftercare process, especially related to post-ICU complications. Some PCPs perceived case management as valuable to obtain a clearer view on patient’s needs. Others felt that they could more effectively conduct these interactions with the patient themselves [73]. PCPs valued their additional education about post-ICU complications [70]: Regular interdisciplinary conferences to promote peer-to-peer learning on patient-tailored and state-of-the-art interventions for patients with the post-intensive-care syndrome (PICS) were discussed as promising by physiotherapists [74].

## Discussion

**Fig. 2:**
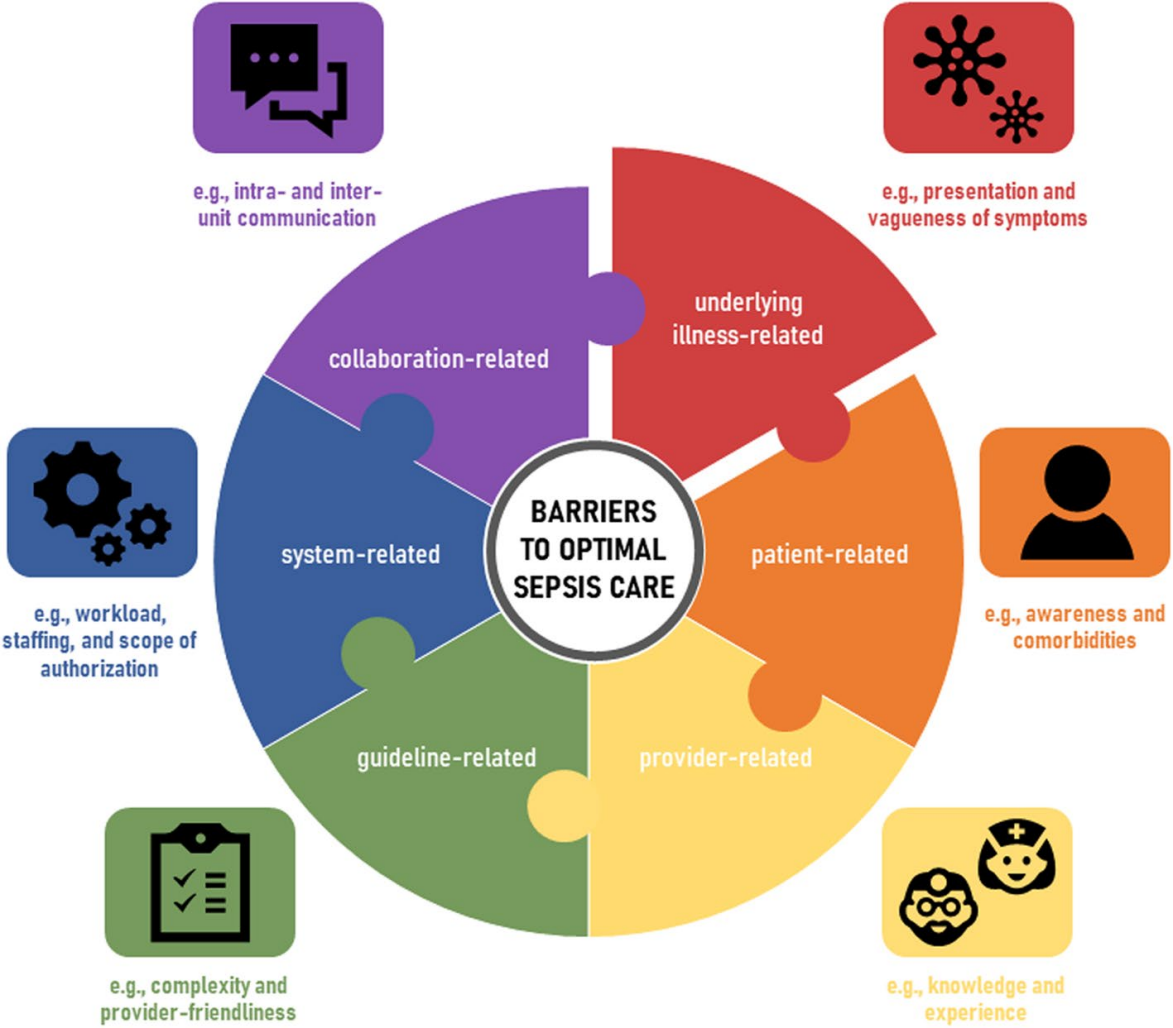
Barriers to optimal sepsis care

This systematized review exploring the perspectives of healthcare providers on sepsis care, uncovered the following barriers and facilitators to successful care that they perceive with regard to (1) prevention, (2) early recognition, (3) timely treatment, (4) transitions of care, and (5) aftercare (Additional file 6). We derived a framework of six interrelated dimensions from the literature for implementing appropriate sepsis care. Good clinical practice and patient safety appear to be underlying illness-related (e.g., presentation and vagueness of symptoms), patient-related (e.g., awareness and comorbidities), provider-related (e.g., knowledge and experience), guideline-related (e.g., complexity and provider-friendliness), system-related (e.g., workload, staffing, and scope of authorization), and collaboration-related (e.g., intra- and inter-unit communication) (Fig. 2). Two aspects generally identified by providers as relevant for improvement are: lack of necessary knowledge on the part of providers and barriers of interdisciplinary and/or intersectoral collaboration and communication.

In general, provider descriptions imply that delayed diagnosis and treatment are related to the patients’ fragmented pathway of care. This fragmentation is characterized by a lack of cross-unit and cross-sectoral communication and suboptimal patient handovers. Furthermore, few hospitals offer subsequent structures as post-ICU clinics, mainly established in the United Kingdom, the USA, and Germany [75–77]. PCPs play a key role in managing the critical illness survivors’ complex web of intertwining impairments. However, they claim to lack the knowledge and expertise necessary to properly manage the needs of this patient group.

Other findings include:

- Knowledge is gained through previous experience.
- Joint discussions with (experienced) colleagues are highly valued.
- Human judgement and intuition are perceived to be complementable but not replaceable by electronic early warning systems.
- Intuitiveness of electronic early warning systems is as important as their performance.
- Clinical intuition is described by professionals as guiding management.
- Of the numerous perceived barriers and facilitators to acute sepsis management only few are within professionals’ control.

While there is a substantial body of literature on the early recognition and acute treatment of sepsis from a provider perspective, there appears to be little research on the follow-up process of sepsis sequelae from a provider perspective. Our search strategy did not identify literature on the perspectives of rehabilitation clinic providers, which implies that the body of literature focusing on this care sector may be understudied. Especially since not only up to 17 to 44% of critical illness survivors report clinically relevant stress symptoms after having received intensive care [78], but also their families commonly develop adverse psychological repercussions referred to as the post-intensive-care syndrome – family (PICS-F) [79], it is also striking that – to our knowledge – there is limited literature on the perspectives of psychotherapists treating the mental sequelae of survivors and their relatives. Findings from previous research signify that an ICU stay may also pose a trauma for relatives, who fear the drastic deterioration and death of their loved ones, possibly to a similar extent as the patients themselves [71, 79], which may affect the abilities of informal caregiving after hospitalization [80].

This review disclosed six potential dimensions (underlying illness, patient, provider, guideline, system, and collaboration) of toeholds to instigate change in sepsis care, some of which are modifiable. As the first dimension, which revolves around the underlying illness, is not something providers or policymakers can have a bearing on, we focus on the practice implications of the remaining five dimensions. With regard to the patient dimension, an international scoping review showed that the public’s sepsis knowledge is generally low [81]. The general unawareness of sepsis as a disease, sepsis definition(s), and its core symptoms are described as disproportionate to the worldwide mortality rate emanating from sepsis. To reduce the number of deaths, the public needs to be educated on how to recognize early symptoms and that prompt medical care is crucial for survival [82]. Mass-mediated health campaigns on various health-related topics have been proven to spark positive effects on constitutional behavior change and knowledge levels [83]. We believe that campaigns that also focus on laypeople and future patients have the power to reduce preventable deaths by raising sepsis awareness and knowledge of what warning signs to look out for and act on. We therefore advocate the consolidation of campaigns aimed at continuous sensitization and education of the general public. However, as awareness and knowledge levels vary from country to country, health campaigns should seek to factor in and address local conditions [81].

Concerning the provider dimension, we recommend measures to improve sepsis knowledge and to counteract not yet acquired work experience, primarily among junior staff. A systematic review [84] summarized that a number of studies illustrate the benefits of sepsis education and training programs’ implementation on provider confidence, knowledge, and skill set in escalating urgent medical management. This is particularly true for programs using active learning approaches, such as simulation and game-based learning, compared with traditional didactic training. Active learning has been shown to produce longer-term effects and to enable improved performance to be maintained over several months [84].

As to issues of guideline conceptualization, our review suggests that the completion of sepsis bundles relies on a complex composition of individual measures, requiring the assistance of multiple staff in an environment that is marked by its busyness and unpredictability. The insufficient compliance with care bundles globally [85, 86] indicates that the collection and grouping of evidence-based measures are not enough to warrant prompt, rapid, and uninterrupted execution. Ethnographic methods can help to critically evaluate and revise existing guidelines and bundles, as they incorporate the realities and multi-layered contexts of real-world practices in place of purely theoretical paradigms [87]. Evidence for sepsis care is often weak and inconsistent, limiting the applicability of guidelines to the individual patient. Sepsis management solely based on guidelines’ generalized actions seems insufficient to treat a patient collective with such a heterogeneity in pathophysiology, clinical implication, and treatment responsiveness. It is assumed that different subsets of septic patients might profit from different interventions. Experts propose personalized sepsis interventions through individualized adaption of guideline recommendations. To this end, the decision to maintain or discontinue a therapeutic intervention should be based on iteratively monitored intervention responses [88]. Personalized immunotherapy might further improve but also complicate sepsis care [89] as individualized sepsis care requires a high level of expertise and clinical experience. In other medical contexts, complicated cases are referred to experts and specialized centers for individualized care. Due to the time-critical nature of the disease and the logistics of critical care transfer, this option is limited in sepsis. Telemedicine might be a possible solution for this. So far, telemedicine has been only assessed for the improvement of standardized care and guideline adherence in sepsis [90, 91].

About system-related issues, conditions of workplaces such as low nurse: patient ratios, high workloads, high nurse turnover levels, staff shortages, and hostile work environment have already been shown to play negatively into patient safety culture [92, 93]. Patient safety describes the prevention of the commission of errors and therefore avoidable patient harm, that occurs in healthcare settings [92]. A landmark report by the Institute of Medicine [94] specified that between 44.000 and 98.000 US citizens die each year as a result of errors that can be traced back to the structure and systemic faults of the healthcare system. These alarming figures have sparked subsequent efforts to reduce the system’s inherent risks to patient safety when delivering care. For instance, the Institute of Medicine [95] published widely cited protective measures to improve patient safety in the work environment of nurses. According to these, practical recommendations can be categorized into eight higher-order policy themes; namely, governing boards that focus on safety, leadership and evidence-based management structures and processes, effective nurse leadership, adequate staffing, organizational support for ongoing learning and decision support, mechanisms that promote interdisciplinary collaboration, work design that promotes safety, and organizational culture that continuously strengthens patients safety. Guiding principles such as these can be used to reform organizational work environments.

Lastly, the work of the Institute of Medicine [95] has already denoted the integral role of interdisciplinary communication and collaboration in improving the delivery of care and hence patient outcomes. Our review implies that efficient communication mechanisms between colleagues, wards, and sectors need to be developed and firmly established. Interprofessional teamwork within and across sectors is vital when dealing with a complex and time-sensitive critical illness, such as sepsis, which requires rapidly initiated and unhindered collaboration and coordination of the responsible care team members. In terms of influences within sectors, reluctance to consult senior staff originating from the fear of being criticized and perceived as unknowing was identified as a factor restricting collaboration. Moreover, whether and when to consult, for instance, medical emergency teams to escalate care for deteriorating patients may be strongly influenced by the primary team physicians’ dominance in patient management [96]. Strategic team-building interventions may be an attempt to improve healthcare providers’ interpersonal relationships between healthcare providers and, consequently, communication and collaboration in acute as well as non-acute healthcare settings [97, 98]. Further research is needed as the effects of team-building interventions on different areas of the perceived work environment (e.g., teamwork attitudes and team functioning) vary across studies [98]. Strategies to improve interprofessional communication processes across sectors, however, appear to be underrepresented in current literature. Notably, patients and survivors describe discontent when providers were unfamiliar with their medical history and therefore unprepared to perform tailored care [99]. For this reason, not only providers but also patients highly appreciate the information flow between providers along the care pathway and acknowledge the associated benefits of the provision of patient-centered care, especially after hospital discharge [99, 100]. Future expert panels should discuss how communication between sectors can be designed as low-threshold, time-efficient, and feasible as possible, while respecting the burden of work that healthcare professionals already have to manage.

### Strengths and limitations

Our aim was to provide a comprehensive overview of the published healthcare professionals‘ perspectives on sepsis care using a robust review methodology. While many of the findings may be familiar to a practicing clinician, our results provide a structured and multidisciplinary framework based on current evidence, that serves as a foundation for further research. This review has strengths. Firstly, the review incorporates a broad spectrum of articles as it considers every phase of the septic patient’s care pathway and the perspectives of all occupational groups involved in sepsis care. Second, critical appraisal has been performed to assess the articles’ relevance and trustworthiness, resulting in an illustration of each item’s evaluation instead of aggregating a single quality score (see Additional files 3, 4, and 5). This illustration serves to counteract the loss of information during the rating process. This review has also limitations. As a systematized review, the literature search was performed in only one electronic database, without independent literature selection by two or more authors and a beforehand published protocol. Additionally, articles that are published in languages other than English and German were not selected for this review and only literature from high-resource settings was included. However, synthesizing literature from countries with a comparably high standard of care, makes the review more pertinent to high-level practice.

## Conclusions

This article reviews literature on providers’ perspectives on sepsis care. A framework of six dimensions for improvement along the complete care pathway (prevention, early recognition, timely treatment, transitions of care, and aftercare) were derived. Most dimensions are beyond professionals’ control, as being related to the underlying illness, the (potentially) septic patient, the guidelines used, the healthcare system, and the collaboration between medical actors. Two main barriers described were the lack of necessary sepsis knowledge and interdisciplinary and/or intersectoral communication. Therefore, future interventions in sepsis care should focus on continuing education and provider development training as well as standardized communication channels across disciplines and sectors. These changes need to be realized at the personal, organizational, and health system levels to mitigate the high personal, medical, and societal burden of sepsis in the future.

## Supplementary Information

Supplementary Material 1.

Supplementary Material 2.

Supplementary Material 3.

Supplementary Material 4.

Supplementary Material 5.

Supplementary Material 6.

## Data Availability

No datasets were generated or analysed during the current study.

## Abbreviations

ACP: Acute care physician
EMS: Emergency medical service
ICU: Intensive care unit
PCP: Primary care provider
QI: Quality improvement
USA: United States of America
