# Supplementary material for: Barriers and facilitators to optimal sepsis care – a systematized review of healthcare professionals’ perspectives
Source: BMC Health Serv Res. 2025 Apr 24;25:591. doi: 10.1186/s12913-025-12777-8 (PMC12020105; doi:10.1186/s12913-025-12777-8)
Supplement: Supplementary file 1 — Supplementary Material 1. [file 12913_2025_12777_MOESM1_ESM.pdf]

Additional file 1

Search string

(sepsis[Title/Abstract] OR septic shock[Title/Abstract) AND (qualitative[Title/Abstract] OR survey*[Title/Abstract] OR questionnaire*[Title/Abstract] OR interview*[Title/Abstract] OR focus group*[Title/Abstract] OR content analys?s[Title/Abstract] OR thematic analys?s[Title/Abstract]) AND (expert*[Title/Abstract] OR professional*[Title/Abstract] OR "Health Personnel"[Mesh]) AND (perspective*[Title/Abstract] OR perception*[Title/Abstract] OR view*[Title/Abstract] OR insight*[Title/Abstract] OR experience*[Title/Abstract] OR opinion*[Title/Abstract] OR attitude*[Title/Abstract] OR barrier*[Title/Abstract]) NOT (newborn*[Title/Abstract] OR neonat*[Title/Abstract] OR pediatri*[Title/Abstract] OR infant*[Title/Abstract] OR child*[Title/Abstract])
