# Supplementary material for: Barriers and facilitators to optimal sepsis care – a systematized review of healthcare professionals’ perspectives
Source: BMC Health Serv Res. 2025 Apr 24;25:591. doi: 10.1186/s12913-025-12777-8 (PMC12020105; doi:10.1186/s12913-025-12777-8)
Supplement: Supplementary file 6 — Supplementary Material 6. [file 12913_2025_12777_MOESM6_ESM.pdf]

Additional file 6: Healthcare professionals' perceived barriers and facilitators to sepsis care

Additional file 6. **Perceived barriers and facilitators to sepsis care.**

**Barriers**	**Facilitators**
**Prevention**	
Knowledge gaps/ uncertainties about risks specific to asplenia<br>Contraindicated concurrent treatments<br>Post-discharge vaccination sequence<br>Updated vaccination recommendations<br>Scarcity of routine care for asplenic patients<br>Misleading information in the discharge letter<br>Hospital's failure to call patients' attention to subsequent primary outpatient care<br>Patients' lack of awareness<br>Patients' comorbidities<br>Patients' concerns about the late effects of vaccination<br>Delivery shortages of vaccines<br>Demanding documentation requirements	
**Early recognition**	
Knowledge gaps in sepsis criteria/ relevant clinical signs<br>Lack of training in detecting sepsis<br>Availability of physicians on the wards<br>Time-limited staff-patient contact<br>Complexity of sepsis<br>Lack of consistency in detecting non-specific warning signs<br>Lack of necessary equipment<br>High patient acuity and volume<br>Lack of insight into patients' baseline status<br>Less qualified junior physicians<br>Lack of supervision by experienced physicians<br>Task-oriented (rather than coaching-oriented) actions	Adherence to blood culture sampling guidelines:<br>• regular training<br>• more time in professionals' work for continuing medical education<br>Ability to seek advice<br>Intercollegiate supervision and reflection<br>Clinical expertise<br>Clinical intuition/ gut feeling<br>Visible cues during direct professional-patient interaction<br>Acceptance/utility of and trust in electronic early warning systems:<br>• when data at hand is diffuse<br>• when the course of action is difficult to predict<br>• when specific interventions are attached to the alert<br>• understanding how the system works

Lack of adherence to guidelines of blood culture sampling:<br>• unawareness of guidelines‘ content<br>• time constraints during everyday work<br><br>Acceptance/utility of and trust in electronic early warning systems:<br>• lack of predictive value<br>• lack of specifity<br>• lack of efficacy<br>• low precision<br>• little patient-centeredness<br>• little transparency<br>• alert fatigue<br>• complexity<br><br>Uncertainties in referral decisions<br><br>Narrowness of guidelines when assessing complex and ambiguous clinical presentations	• direct experience with the system<br>• external studies validating its effectiveness<br>• recommendations from colleagues and experts<br>• integration of own recommendations into the tools‘ operation<br>• minimization of the alert frequency by optimizing the threshold<br>• provision of explanations of the alert content<br>• integration of the alert into the workflow<br>• provision of direct feedback<br>• interventions that are based on an established treatment protocol<br>• interventions that focus on the patient’s clinical condition as a whole rather than on predefined thresholds<br>• indication to call the medical emergency team<br><br>General patient appearance<br><br>Patient history<br><br>Physical examination of the patient<br><br>Previous experiences with similar patient cases
**Timely treatment**	
Rather low prioritization of sepsis medical initiative compared to other medical initiatives<br><br>Communication of sepsis-related health risks to patients<br><br>Uncertainties about whether the patient needs fluid<br><br>Uncertainties about the appropriate fluid volume<br><br>Lack of research and evidence on prehospital fluid therapy<br><br>Poor adherence to protocols outside the ICU / by specialties outside of anesthesia:<br>• lack of knowledge<br>• complexity of existing protocols with sequential and interdependent steps of coordination, collaboration, and communication among multidisciplinary staff<br>• non-intuitiveness<br>• resource-intensiveness<br>• information-overload<br>• provider-unfriendliness<br>• unmet needs of individual patients<br>• heavy clinical documentation<br><br>Complexity and vagueness of sepsis manifestation	Clinical intuition<br><br>Use of sepsis screening tools and protocols<br><br>Schooled response team (prompt and uninterrupted escalation of care with less coordination and task-switching)<br><br>Granting nurse-initiated procedures without physician approval and prescription<br><br>Knowledge of sepsis triggers<br><br>Familiarity with the respective protocols/bundles<br><br>Education and training on how to handle non-standard situations<br><br>Perceived effectiveness of the respective protocols<br><br>Perception that benefits of care escalation outweigh potential risks<br><br>Feedback, support, and advice from experienced colleagues<br><br>Transdisciplinary collaboration in the ICU<br><br>High probability of infection<br><br>High illness severity<br><br>Clinicians‘ specialty (emergency medicine)

High resource demands of sepsis care:<br>• time pressure<br>• busy/heavy workload<br>• high patient acuity<br>• competing demands<br>• interruptions in workflow<br>• staffing shortages<br>• delays in interventions by nurses<br>• equipment/treatment unavailability<br>• time-consuming patient transfers<br><br>Knowledge gaps (e.g., antibiotic pharmacokinetics and pharmacodynamics)<br><br>Low level of experience<br><br>Low level of assertiveness<br><br>Low level of training<br><br>Small range of clinical skills<br><br>Low provider engagement<br><br>Negative attitudes towards protocol use<br><br>Fear of harming the patient in the absence of an established diagnosis<br><br>Fear of septic patients in general<br><br>Insufficient prioritization of treatment of severe sepsis and septic shock<br><br>Nurses' lack of authorization (dependence on physician prescriptions and orders)<br><br>Uncertainty about the chain of command<br><br>Physician unavailability<br><br>Delays in prescriptions<br><br>Delays in laboratory results and other diagnostics<br><br>Rather task-oriented and less holistic assessment → reduced critical thinking and clinical reasoning<br><br>Imbalance in initiating but not reviewing/stopping infection management for junior physicians<br><br>Lack of feedback to junior physicians from experienced senior physicians → little learning gains in infection management<br><br>Low probability of infection<br><br>Low illness severity<br><br>Clinicians' specialty (other than emergency medicine)<br><br>Lack of/ inadequate availability of antibiotics on the unit	High clinical experience

Lack of awareness that intravenous antibiotics were available on/ ordered to the unit<br><br>Intravenous line access issues	
**Transitions of care**	
Failures in communication and collaboration among providers:<br>• hostile/intimidating work environments characterized by little respect<br>• interdisciplinary conflicts<br>• poorly coordinated patient handovers to another unit or sector<br>• medical histories and anamneses with incorrect content<br>• absence of (the responsible) physician(s)<br>• lack of information about the urgency with which to treat septic patients<br><br>Low capacity of available hospital beds<br><br>Lack of staff for transportation<br><br>Long latencies of laboratory results and/or other diagnostics<br><br>Different heterogeneous documentation systems	Mandatory training sessions<br><br>Guiding checklists and posters<br><br>Reviews of septic patient cases who received substandard care<br><br>Establishment of standard operating procedures<br><br>Experienced and knowledgeable physician undertaking emergency department triage<br><br>A physician as a central point of contact for all wards<br><br>Presence of a physician at the point of transition<br><br>Completion of a sepsis checklist for all newly admitted patients<br><br>Personal verbal instructions<br><br>Feedback to facilitate reflective learning<br><br>Effective communication pathways<br><br>Early warning scores<br><br>Revised electronic templates for recording physiologic parameters<br><br>Education about sepsis<br><br>Completion of an electronic summary of patients' clinical and social information compiled by the patient's PCP and made available to other medical parties<br><br>In-hospital quality improvement team
**Aftercare**	
Little communication about sepsis sequelae and consequences with survivors, their families, and PCPs<br><br>Limited and incomplete information sharing between ACPs and PCPs (e.g., by telephone, discharge summary) due to:<br>• time constraints<br>• perception of low priority<br>• difficulties in establishing contact with the PCP<br><br>Reception of lay clinical information by patients and relatives<br><br>Absence of care after hospital discharge	Two-way information flow between ACPs and PCPs when:<br>• unplanned admission to the ICU<br>• unplanned discharge from the ICU<br>• patient death in the ICU<br><br>Brief information sharing at ICU admission<br><br>More explicit provision of information at discharge about:<br>• why the admission occurred<br>• what the consequences were<br>• what to target in future treatment<br><br>Contact at ICU admission (between ACPs and PCPs) should not only serve to inform the PCP

Direct collaboration of different disciplines in the fragmented pathways of care<br><br>PCPs‘ limited time, knowledge, experience, and skills to adequately support patients in the aftercare process<br><br>Lack of availability of and access to further supportive care services (e.g., physiotherapy and psychotherapy)<br><br>Health insurance issues and limited cost coverage	about the critical illness but also to provide ACPs with patient data relevant to early patient management<br><br>Care coordination between inpatient to outpatient clinic services that offer:<br>• diagnostics<br>• counseling<br>• psychological aftercare<br>• targeted referral to specialists<br>• relatives’ involvement<br><br>PCPs‘ continuity of care and their good relationship with patients, characterized by detailed knowledge of patients‘:<br>• medical history<br>• social background<br>• personality traits<br>• illness coping mechanisms<br><br>Filling patient education gaps, especially related to post-ICU complications → patient engagement<br><br>Case management<br><br>PCPs‘ education about post-ICU complications<br><br>Regular interdisciplinary conferences to promote peer-to-peer learning
